# Thyroid Cancer and Circadian Clock Disruption

**DOI:** 10.3390/cancers12113109

**Published:** 2020-10-24

**Authors:** Roberta Malaguarnera, Caterina Ledda, Agnese Filippello, Francesco Frasca, Vincenzo Cristian Francavilla, Tiziana Ramaci, Maria Chiara Parisi, Venerando Rapisarda, Salvatore Piro

**Affiliations:** 1 School of Human and Social Sciences, “Kore” University of Enna, 94100 Enna, Italy; roberta.malaguarnera@unikore.it (R.M.); vincenzo.francavilla@unikore.it (V.C.F.); tiziana.ramaci@unikore.it (T.R.); mariachiara.parisi@unikore.it (M.C.P.); 2 Department of Clinical and Experimental Medicine, Occupational Medicine, University of Catania, 95100 Catania, Italy; vrapisarda@unict.it; 3 Department of Clinical and Experimental Medicine, Internal Medicine, Garibaldi-Nesima Hospital, University of Catania, 95122 Catania, Italy; agnese.filippello@gmail.com (A.F.); salvatore.piro@unict.it (S.P.); 4 Endocrinology Unit, Department of Clinical and Experimental Medicine, Garibaldi-Nesima Hospital, University of Catania, 95122 Catania, Italy; f.frasca@unict.it

**Keywords:** thyroid cancer, clock genes, machinery, circadian misalignment, sleep disturbances, insulin resistance, occupational and environmental factors

## Abstract

**Simple Summary:**

In this manuscript we review the recent literature supporting a biological link between circadian clock disruption and thyroid cancer development and progression. After a brief description of the involvement of the circadian clock machinery in the cell cycle, stemness and cancer, we discuss the scientific evidence supporting the contribution of circadian clockwork dysfunction in thyroid tumorigenesis and the possible molecular mechanisms underlying this relationship. We also point out the potential clinical implications of this link highlighting its impact on thyroid cancer prevention, diagnosis and therapy.

**Abstract:**

Thyroid cancer (TC) represents the most common malignancy of the endocrine system, with an increased incidence across continents attributable to both improvement of diagnostic procedures and environmental factors. Among the modifiable risk factors, insulin resistance might influence the development of TC. A relationship between circadian clock machinery disfunction and TC has recently been proposed. The circadian clock machinery comprises a set of rhythmically expressed genes responsible for circadian rhythms. Perturbation of this system contributes to the development of pathological states such as cancer. Several clock genes have been found deregulated upon thyroid nodule malignant transformation. The molecular mechanisms linking circadian clock disruption and TC are still unknown but could include insulin resistance. Circadian misalignment occurring during shift work, jet lag, high fat food intake, is associated with increased insulin resistance. This metabolic alteration, in turn, is associated with a well-known risk factor for TC i.e., hyperthyrotropinemia, which could also be induced by sleep disturbances. In this review, we describe the mechanisms controlling the circadian clock function and its involvement in the cell cycle, stemness and cancer. Moreover, we discuss the evidence supporting the link between circadian clockwork disruption and TC development/progression, highlighting its potential implications for TC prevention, diagnosis and therapy.

## 1. Introduction

Thyroid cancer (TC) represents the most common endocrine malignancy, which has shown a strikingly increasing incidence over the past few decades [1,2]. Well-differentiated TC histotypes, comprising papillary TCs (PTCs, 85%) and follicular TCs (FTCs, 15%) account for the majority of TCs and are considered to be low risk tumors [3]. Poorly differentiated TCs (PDTCs) and anaplastic TCs (ATCs) are less common but more aggressive histotypes, often unresponsive to conventional treatments [3].

Epidemiological studies have suggested that environmental factors and lifestyle modifications can be responsible for the increased incidence of TC worldwide. Among the potential modifiable risk factors of TC, particular attention has been paid to insulin resistance and hyperinsulinemia [4]. These metabolic alterations have also been rapidly increasing worldwide due to lifestyle modifications, which may also include circadian clock disruption. At present, it is not completely clear how insulin resistance and related metabolic disorders may affect well-known molecular pathways involved in the pathogenesis of TC such as MAPK, PI3K/PTEN/AKT, TSH-R, and mTOR/p70S6K. To date, many etiopathogenetic features of TC still remain unknown.

Recently a relationship between the circadian clock machinery function and TC has been proposed [5,6,7,8,9,10]. During evolution, organisms have developed biological clocks to better adapt to various rhythmic events such as daily and seasonal fluctuations. Circadian rhythms are generated by a central clock located in the brain’s suprachiasmatic nuclei and by multiple peripheral cellular clocks [11]. A 24 h cell-autonomous circadian clock, virtually present in all cells of the body, regulates several physiological functions, including endocrine rhythms [12].

Disruption of the circadian timing system caused by circadian misalignment such as shift work, chronic jet lag, high fat intake, inappropriate eating times, and abnormal sleep patterns could be responsible of insulin resistance, diabetes mellitus type 2, obesity, metabolic syndrome, cardiovascular diseases and several types of cancers, including TC [13,14,15]. Conversely, proper coordination of circadian behavior and sleep homeostasis may improve several conditions including insulin resistance and overall metabolic fitness [16,17]. The molecular mechanisms linking circadian clock disruption and TC are still unknown but could be, at least in part, insulin resistance. Indeed, this metabolic alteration is associated with a well-known risk factor for TC i.e., hyperthyrotropinemia [18,19,20] which, in turn, has also been associated to sleep disturbances [21]. Alterations in the rhythmicity of thyroid stimulating hormone (TSH) secretion and hypothalamic-pituitary-thyroid (HPT) axis function as well as modifications in genes controlling the cell cycle, apoptosis, DNA damage, inflammation, and immune response are the main mechanisms proposed to mediate circadian-related thyroid disorders [22,23]. Furthermore, variants of various clock genes (*PER2–3, CRYs, BMAL1, REV-ERBs* and *RORs*) and strong changes in their expression profile have been found on thyroid nodule malignant transformation and have been proposed as potential biomarkers for thyroid nodule pre-operative diagnostics [5,7,8]. Although at present fine-needle aspiration biopsy (FNA) represents the gold standard for the preoperative diagnosis of TC, 20–30% of lesions are indeterminate based on cytological features [3,24]. However, FNA is unable to distinguish between follicular adenoma and follicular carcinoma [25,26,27,28,29]. Molecular testing of FNA samples is a new strategy that can help to rule in or rule out the diagnosis of TC, to reduce the use of diagnostic surgery and to better define the prognosis. Genomic studies of differentiated TC have demonstrated that the most recurrently altered genes are *BRAF^V600E^*, *RAS* and *RET/PTC* [30]. Over the last several years new molecular alterations (such as gene fusions, copy number variations, driver mutations, indels, abnormal gene expression, miRNAs) either entirely novel in this cancer or novel alterations of known drivers have been identified [30,31,32]. Some of these molecular markers sometimes coexist with *BRAF* or *RAS* mutation influencing fundamental aspects of TC phenotype and its biological behavior. For example, the combination of *TERT* mutation and a *BRAF* or *RAS* mutation within the same tumor is associated with low degree of differentiation, aggressive behavior and high risk of recurrence and mortality [33]. Yet, it has been demonstrated that *BRAF^V600E^* PCTs represent a spectrum of tumors consisting of at least four distinct molecular subtypes with different genomic, epigenomic and proteomic profiles, suggesting the presence of molecular diversity among PTCs [30,32]. At present, the available molecular tests of FNA samples (ThyroSeq version 3, Afirma Genomic Sequencing Classifier, ThyGeNEXT/ThyraMIR and ThyroPrint) allow one to detect a broad spectrum of molecular alterations [34,35]. Despite the efforts that have been made over the last decade, the diagnostic and prognostic performance of these molecular approaches and their applicability in the routine diagnostic laboratory for thyroid nodules, especially those with indeterminate cytology, are poor and require further validation [36]. Expanding the existing tests by incorporating further reliable preoperative markers predictive of malignancy for suspicious or indeterminate thyroid nodules and/or of disease progression, in combination with the already known molecular alterations and clinical examination, would enhance the diagnostic performance of molecular testing and could have great clinical importance.

In this review, we explore the relationship between disrupted circadian clock machinery and TC. We first describe the mechanisms controlling the circadian clock functions and its involvement in the cell cycle, stemness and cancer. The molecular mechanisms underlying thyroid tumorigenesis will then be summarized. Finally, the scientific evidence supporting the possible biological link between the disruption of circadian clockwork and TC development/progression and its potential role for the TC prevention and treatment will be discussed.

## 2. Circadian Clock

### 2.1. Regulation

All living organisms have developed an internal circadian system oscillating within a period of roughly 24 h in order to adapt to environmental cues. This system is composed of two components: a central master clock and peripheral clocks, all of which are developmentally regulated [11]. The central clock is located in the anterior hypothalamic suprachiasmatic nucleus (SCN), as suggested by the observation that SCN lesions disrupt circadian rhythm, while SCN transplantation restores it [37,38]. The SCN is composed of thousands of neurons, which contain a cell-autonomous circadian clock with a specific rhythm [39,40]. The SCN entrains to environmental light-dark cycles sending signals to the peripheral clocks of each tissue and of almost all of the cells in the body [41] to control rhythms in physiology, metabolism, behavior, immune, hormonal and neural functions [12].

**Figure 1:**
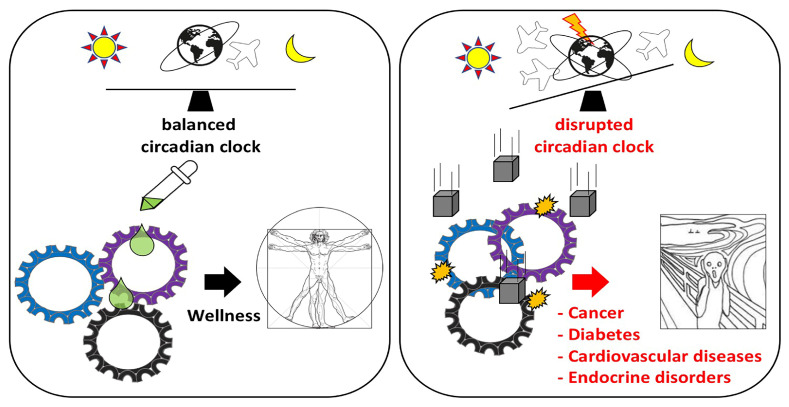
Effect of circadian rhythm disruption on body health. Circadian alignment is associated with wellness and body health. Circadian clock malfunctioning induced by genetic factors (clock gene mutations) and/or environmental factors (inappropriate light exposure, sleep restriction, jetlag, shift work, irregular food intake) can lead to the development of several disorders including cancer, diabetes, cardiovascular disorders, endocrine diseases, inflammation, mental disorders, immune system alterations and reproductive disorders.

At the molecular level, the core pacemaker of each clock is regulated by a set of genes named “clock genes”, which control the cycling of mRNAs and proteins, called “clock-controlled genes (*CCGs*)”, through positive or negative transcriptional/post-transcriptional feedback loops [42]. Many *CCGs* are involved in important physiological and pathophysiological networks and signaling pathways regulating tissue and organ functions. Therefore, the disruption of the circadian clocks in the body could contribute to develop different pathological conditions. Indeed, shift workers who live in a chronic state of circadian misalignment, show an increased prevalence of many diseases including insulin resistance, cardiovascular disorders, gastrointestinal disturbances, depression, neurological alterations, and cancer [11,43] (Figure 1).

In mammals, the circadian clock molecular machinery includes several genes, among which are the following: *CLOCK, BMAL1, NPAS2, Per1-2-3, CRY1-2, DEC1-2, REV-ERBα, RORα, CK1ε, CK1δ* and *TIM* [40,41,44]. The central elements are represented by *BMAL1, CLOCK* and *NPAS2*. They form the positive control loop of the circadian clock. In the nucleus, BMAL1/CLOCK and BMAL1/NPAS2 heteromize and activate the transcription of other clock genes such as *CRY, PER* and *DEC*, which translocate to the cytoplasm. In turn, phosphorylated PERs/CRYs cytoplasmic heterodimers are transported back into the nucleus. PER and CRY proteins, present in the nucleus, inactivate BMAL1/CLOCK and BMAL1/NPAS2 complexes repressing their own transcription as well as the transcription of *DEC1-2* and *REV-ERBα-RORα,* thereby closing a negative feedback loop [45,46,47]. *DEC1* and *DEC2*, by binding to the regulatory DNA core enhancer sequences “CANNTG” of their promoter, directly inhibit their own transcription [48]. *RORα* and *REV-ERBα* constitute a supplementary loop, which acts through RORE elements present in the *BMAL1* promoter to activate or inactivate *BMAL1* transcription, respectively. A further modulation of the nucleocytoplasmic shuttling of all these core clock components is represented by the protein kinases CK1ε and CK1δ, which phosphorylate elements belonging to both positive and negative loops [49,50]. Post-translational and transcriptional modifications such as acetylation, methylation, SUMOylation and ubiquitination contribute to regulate the oscillating of the clockwork circuitry [51,52].

### 2.2. Circadian Clock and Cell Cycle

Recent evidence has highlighted a connection between the circadian clock and cell cycle machinery in healthy and pathological states. The physiological circadian-dependent regulation of cell cycle phases is suggested by the observation that cell cycle progression occurs at specific times of the day/night rhythm [53]. Furthermore, several proteins controlling G1/S and G2/M phases as well as checkpoints involved in DNA repair after damage are rhythmically expressed and regulated by *CCGs* [54,55]. For instance, *P21 WAF1/CIP1*, a negative regulator of G1/S phase progression, is alternatively activated or repressed by RORα and REV-ERBα, respectively [56]. These two proteins bind the same RORE element in the *P21* promoter leading to the activation or inhibition of the CDK2/Cyclin E complex and, consequently, G1/S progression. The expression of another component of cell cycle machinery, CyclinD1, is indirectly regulated by *PER1* and *PER2* genes by inhibiting the transcription of c-MYC. In fact, PER1-2 ablation abolishes c-MYC repression, resulting in elevated cyclin D1 expression, G1/S progression and, therefore, cell proliferation [57]. In contrast, overexpression of PER2 induces cell cycle arrest [58]. PER2 is also involved in the regulation of p53 stability [59,60]. PER2 directly associates with p53 and with its negative regulator MDM-2. The formation of this trimeric complex in the nucleus impairs MDM2-mediated ubiquitination and degradation of p53, resulting in p53 stabilization. On the other hand, p53, acting as a direct competitor of the *BMAL1/CLOCK* binding to *PER2* promoter, represses *PER2* gene expression [61]. However, high levels of BMAL1/CLOCK or BMAL1/NPAS2 activate the expression of the tyrosine kinase WEE1, which inhibits CDK1/Cyclin B complex and represses G2/M transition. Conversely, CRYs repress WEE1, favoring cell proliferation [55]. PER1 and TIM, by acting as co-factors or adaptor proteins, lead to the activation of Ataxia Telangiectasia Mutated (ATM) or Ataxia Telangiectasia and Rad3-related protein (ATR) [18,54,62], which in turn activate Checkpoint kinase 1 (CHK1) and Checkpoint kinase 2 (CHK2). Phosphorylated CHK1 and CHK2 are responsible for cell cycle arrest and apoptosis by the inactivation of CDKs [63,64]. All these molecular interactions may represent a regulatory link between the cell cycle, p53-mediated cellular damage response and the circadian clock-regulated cellular pathways. The disruption of cell cycle regulation as a consequence of circadian clock rhythm perturbation could lead to uncontrolled cell division and, consequently, to the development of cancer.

### 2.3. Tumor Suppressor or Oncogene: The Janus Face of the Circadian Clock Machinery

Circadian clock function and cancer are interlinked. The synchronized circadian clock is an important tumor suppressor, while disruption of clock genes affects tumor development and cancer susceptibility [65,66,67,68]. Although several in vitro and in vivo studies support this observation, the molecular connections and the relationship between clockwork and cancer are still not well understood and remain controversial [69]. For instance, *PER1* and *PER2* behave as tumor suppressors in vivo [57]. Mice bearing the *PER2* mutation and lacking circadian rhythm show increased incidence of malignant lymphomas and an increased rate of mortality after ionizing radiation relative to wild-type controls. This tumor promoting effect is likely due to decreased BMAL1 expression and consequent increased c-MYC expression [57,70]. However, other findings have shown that deficiency in *PER* genes (*PER1* or *PER2*) has no effect on the rate of spontaneous and radiation-induced carcinogenesis [71].

Conversely, PER2 overexpression causes growth inhibition, apoptosis and cell cycle arrest in different cancer cell models [72,73,74]. Altered expression of PER1, PER2 and/or PER3 have been reported in colorectal, pancreatic, gastric, oral, breast, prostate, bladder, renal, and non-small cell lung cancers, as well as in glioma, hepatocellular carcinoma, head and neck squamous cell carcinoma and myeloid leukemia [75,76,77,78,79,80,81,82,83,84,85,86,87].

With respect to the other components of the core clock, *CRY* mutant mice lacking circadian rhythm [88] have a faster rate of implanted tumor growth, more susceptibility to ionizing radiation-induced cancer, and increased morbidity and mortality, likely due to defective cell cycle checkpoints and DNA repair ability [89,90]. However, the increased predisposition of arrhythmic *CRY−/−* mice to spontaneous and DNA damage-induced cancers has not been confirmed by other studies. Gauger et al. have showed that *CRY* double knockout (DKO) mice behave similarly to wild-type controls with respect to spontaneous and radiation-induced morbidity, mortality and cancer [70]. Similarly, fibroblasts derived from the *CRY* mutant mice have the same sensitivity to ionizing and UV radiations and the same cellular response to DNA damage, compared to wild-type control fibroblasts [70]. On the other hand, later studies demonstrated that *CRY1−/−*; * CRY2−/−* deficient mice in a *p53−/−* background showed an increased survival and protection from tumor development [91]. However, *CRY* mutation makes *RAS*-transformed *p53* null cells, but not *p53* wild type cells, more susceptible to apoptosis [92,93].

Unlike *CRY* DKO mice, loss of *CRY2* alone induces increased tumor burden and enhanced susceptibility to transformation [94], supporting an unexpected function of *CRY2* in contributing to circadian protection from tumor formation.

Furthermore, recently it has been demonstrated that *CRY1* and *CRY2* exert opposite roles in modulating transcription of several factors, such as c-MYC, in response to DNA damage [95]. The discrepancies observed among various studies may be attributable to several reasons: the real divergent roles of *CRY1* and *CRY2*; the different genetic backgrounds of mice; the severity of the circadian clock disruption caused by *CRY* knockout; the establishment of homeostatic mechanisms; the cooperation between *CRY2* deficiency and multiple oncogenes in the control of proliferation and transformation.

The other central component of clock machinery, *BMAL1*, has notoriously been considered a tumor suppressor gene. However, as seen for other circadian clock genes, there are different findings from different laboratories showing both pro- and anti-cancer effects of *BMAL1* KO mutation. Some studies have demonstrated that downregulation of *BMAL1* gene expression promotes cancer cell proliferation, invasion, and tumor growth and decreases apoptosis induced by DNA damage [96,97,98]. Conversely, BMAL1 overexpression has been seen to inhibit cell proliferation, invasiveness and to increase sensitivity to anticancer drugs [99,100,101]. In support of the anticancer effect of this clock gene, whole-body or organ specific KO of *BMAL1* in mice has been associated with increased lung cancer and hepatocellular carcinoma [102,103]. In contrast to these data, *BMAL1* KO has been found to suppress proliferation and anchorage-dependent and independent clonal growth of malignant pleural mesothelioma cells [104]. Similarly, *BMAL1* KO decreases apoptosis of murine colon cancer cells and fibroblast cells in response to chemotherapeutic drugs [98]. However, a study by Puram et al. has shown that genetic deletion of *BMAL1* results in suppression of leukemia formation [105]. The opposite and divergent effects of *BMAL1* on carcinogenetic mechanisms have recently been confirmed in untransformed MCF10A and in invasive MDA-MB231 breast epithelial cell lines. In these cellular models, *BMAL1* deletion by CRISPR technology induced apoptosis in response to genotoxic agents but at the same time increased the invasive potential of MDA-MB231 cells. Altogether these results suggest that *BMAL1* may exert both protective and pro-tumor effects based on the different cellular contexts and on the activation of circadian dependent or independent functions of the *BMAL1* gene in different organs [106].

Similar to *BMAL1*, studies on the role of the *CLOCK* gene in carcinogenesis have often been contradictory. A study by Lee et al. [89] found that *CLOCK* Δ19/ Δ19 mice had enhanced tumorigenesis under basal and irradiated conditions in contrast to other studies showing that *CLOCK* gene deletion in mice did not increase the incidence of cancer [54,71]. In support of the pro-tumor role of the *CLOCK* gene, other evidence found that *CLOCK* knocking-down decreased cancer proliferation, progression and invasion as well as expression of several cancer-associated genes [107,108]. These pro-tumor effects of the *CLOCK* gene are likely due to its transcriptional functions as well as to its intrinsic histone acetyltransferase (HAT) activity [109]. Through this HAT activity, *CLOCK* may play a pivotal role in chromatin remodeling and in modulating the activity and the transcription of proteins involved in cell cycle control and DNA damage response, thereby influencing cancer development [110]. For example, in breast cancer, *CLOCK* may modulate estrogen receptor-α mediated gene expression using its HAT activity [110].

Epidemiological evidence supports the possibility that disruption of the circadian clock periodicity may be implicated in increased cancer risk and in the progression of the disease [111,112]. As suggested by different independent studies and meta-analyses, night workers, shift workers or people often subjected to jet lag or to prolonged light exposure during the night, present an increased incidence of breast [113,114,115,116,117,118,119,120,121,122,123,124,125,126,127,128], prostate [129,130,131,132,133,134,135,136,137,138,139], colon [40,140,141] and endometrial epithelial cancers [142], as well as non-Hodgkin’s lymphoma [143]. Furthermore, cancer patients with altered circadian rhythm have poorer survival compared to those with normal circadian clock periodicity [144]. All these epidemiological studies strongly suggest that the lack of circadian rhythm homeostasis contributes to cancer risk, cancer development and progression. In light of these results and based on sufficient evidence from experimental animal models, the Agency for Research on Cancer has classified “shift work with circadian clock desynchrony” as a potential carcinogenic to humans (group 2A) [145,146].

Several plausible hypotheses have been proposed to explain the link between circadian clock disruption and cancer, among them: the suppression of nocturnal peak of melatonin after exposure to light at night; immune system alterations as a consequence of sleep deprivation; shift in the ratio between anti-tumor and pro-tumor cytokines, induction of inflammation response, modifications in the levels of appetite-regulating hormones, internal desynchronization and disturbances in the regulation of several clock genes controlling the cell cycle, apoptosis, DNA damage repair and cell proliferation. However, further studies are needed to better investigate the different day/night alternation systems, sleep patterns, chronotypes, measurement of biomarkers, presence of polymorphisms or other abnormalities in clock genes in order to discover new potential prognostic markers and novel therapeutic targets for specific cancers [66,139,147,148].

### 2.4. Circadian Clock and Stemness

A large body of evidence has shown that the circadian clock influences stem cell biology, lineage commitment, tissue regeneration and aging [149]. The core of the clock machinery, including *CLOCK* and *BMAL1* genes, is common to different organs and tissues, while the resulting rhythmic and phased transcription of peripheral output clock genes controlled by the central core circuitry is highly tissue-specific. The functional integrity of both central and peripheral clocks and the tissue-specific gene expression programs meet the physiological needs of every organ, thereby ensuring tissue homeostasis and adaptation to the circadian rhythm of the environment. Perturbation of physiological circadian clock equilibrium has been implicated in several processes of tumorigenesis, even at early stages of its development [149,150,151,152].

In vitro and in vivo studies have demonstrated that regulation of circadian clock programs is different in pluripotent stem cells, adult stem cells and differentiating cells. Pluripotent embryonic stem cells (ES), although expressing most of the clock genes at low levels, lack a rhythmic clock system [150,153,154]. The diurnal oscillatory network starts to be gradually activated during the differentiation process [149,150]. Conversely, reversing differentiation through reprogramming processes decreases rhythmicity of the expression of clock-related genes [154]. It is still unknown whether clock factors expressed in ES exert a role in stem cell maintenance. *BMAL1, CLOCK*, and *PER2* KO mice are not embryonically lethal [11,155,156] but they show premature aging and age-related diseases [155]. As suggested by Dierickx et al., it is plausible that the different level of clock factor expression at embryonic stages compared to differentiated cells might exert an unrelated clock function during embryonic development, which becomes important and prevalent at later stages in life [149]. Adult stem cells, unlike ES, possess a functional circadian clock [152], which guarantees stem cell proliferation and self-renewal, thereby facilitating tissue homeostasis, regeneration and a stress-associated response [157].

In fact, disruption of the clock components *PERs, CLOCK* and *BMAL1* has been shown to affect regulation of hair follicle bulge stem cell cycling [158], cell-intrinsic keratinocyte differentiation or proliferation responses [159,160,161,162], epidermal wound repair [163], myocardial response to infarction [164], lung response to pro-inflammatory cues [165], hematopoietic system replenishment [166,167], intestinal stem cell renewal and intestinal epithelial regeneration especially after damage from gastrointestinal disease [168,169,170,171].

Dysregulation of the circadian network has also been implicated in cancer stem cell biology.

Targeting BMAL1/CLOCK machinery using small molecule agonists of CRY and REV-ERB, induced a synergistic anti-proliferative effect in glioma stem cells (GSCs) [172]. The oncogenic role for circadian clock activity in the cancer stem cell compartment has been confirmed by other observations. For instance, PER2 mRNA and protein expression was down-regulated in glioma stem cells (GSCs) compared to non-stem glioma cells, while PER2 overexpression induced GSC cell cycle arrest at the G0/G1 phase and suppression of proliferation, a stem cell-like phenotype and invasion capability by targeting the Wnt/β-catenin signaling pathway [173]. However, PER1/2 expression correlates with WHO grading of glioma, being downregulated in glioma tissue compared to normal brain tissue [85]. All these findings suggest that the *PER2* gene exerts a potential role in regulating stemness, self-renewal, cell growth, cell cycle distribution, migration and invasion of GCS in glioma and are consistent with similar results obtained in colon cancer stem-like cells (CCSCs). In this cell subtype, PER overexpression inhibits self-renewal properties and chemo-resistance via downregulation of β-catenin and NOTCH signaling pathways [174]. Involvement of the core circadian clock genes in stemness has also been demonstrated in other CSC contexts such as myeloid leukemia stem cells [105], breast cancer stem cells [175] and in the initial steps of hepatocarcinogenesis [102]. Despite this evidence, some aspects and control mechanisms of stem/progenitor cell biology by clock machinery still remain unknown also because they may be influenced by the cellular context, tumor development and differentiation stages. However, on the basis of the data present in the literature to date, targeting one or more components of the circadian machinery could represent a new opportunity for the development of novel anti-cancer therapies.

## 3. Thyroid Tumorigenesis

Based on the cell of origin, TC can be divided into two main categories: follicular epithelial cell-derived carcinomas (>95%) and medullary TC (3–5%) arising from C cells. Tumor arising from follicular epithelial cells include papillary TC (PTC), follicular TC (FTC), Hurthle cell carcinoma (HCC), poorly differentiated TC (PDTC) and anaplastic TC (ATC). The last two tumor subtypes are very rare but more aggressive follicular-derived TCs compared to differentiated TCs [1]. Recently, integrated genomic, transcriptomic, proteomic and miRNA analysis has been developed to better examine the molecular mechanisms responsible of the different structural features and behaviors between the different TC subtypes. Thyroid tumorigenesis classically occurs through a multistep dedifferentiation process, which starts from well-differentiated TCs and proceeds through poorly differentiated to anaplastic carcinoma. According to this model of tumorigenesis, constitutional activation of the MAPK signaling pathway via *RAS, BRAF* mutations and/or *RET/PTC* rearrangements and Paired-box gene 8/Peroxisome Proliferator-Activated Receptor gamma *(PAX8/PPARγ )* fusion transmit growth signals to normal thyrocytes, thereby playing a driver role in their malignant transformation. The most common molecular alteration includes the mutation in the *BRAF* gene, which appears activated in 35–60% of PTCs [176]. Rearrangements of *RET* gene *(RET/PTC)* (especially *RET/PTC1* and *RET/PTC3*) are specific molecular alterations present in 5% to 30% of PTCs [176]. The follicular variant of PTCs usually harbor *RAS* mutations or *PAX-8/PPAR-γ* translocations [177,178,179]. However, several other molecular alterations including abnormal gene expression, point mutations, copy number changes, gene fusions in components of other survival-signaling cascades, such as TSH-R, PI3-K/Akt, mTOR, and the IGF pathways have been identified as potential contributors to TC development and progression [180,181,182,183,184,185]. For instance, roughly 40% of well differentiated TCs and more than 50% of highly aggressive TCs carry *PTEN* downregulation or gene silencing [186]. Point mutations or copy number alterations of *PIK3CA* and *Protein Kinase B* (*PKB* also known as *AKT*) are present in ~23% of ATCs sometimes coexisting with either *RAS* or *BRAF* mutations [185]. A proportion of TCs, showing an aggressive behavior, often overexpress components of the IGF system such as insulin receptor isoform A (IR-A), insulin-like growth factor-2 (IGF-2) and insulin-like growth factor-1 receptor (IGF-1R) [187]. Indeed, overexpression of IR-A and the activation of IR-A/IGF-2 loop is a feature of PDTCs, ATCs or stem-like TC cells [188,189] and it is associated to resistance to some targeted therapies [190,191]. The functional interactions between the IGF system and other molecules, such as the non-integrin collagen receptor discoidin domain receptor 1 (DDR1) and the receptor for the hepatocyte growth factor (HGF) MET, may amplify the biological response to insulin, insulin-like growth factors (IGFs), and HGF contributing to favor TC initiation, progression, de-differentiation and metastatic features [192,193,194,195,196,197,198,199,200]. Much evidence has suggested that overactivation of the IR/insulin axis, present in different metabolic disorders characterized by insulin resistance and hyperinsulinemia, plays a putative role in TC tumorigenesis being associated with TC increased risk and worse prognosis [4,201]. In addition, mutations in *p53* family members, *TERT* promoter, *ATM, RB1, MEN1, NF1, NF2, SWI/SNF*, mismatch repair genes, and histone methyltransferase have been associated with tumor de-differentiation process and tumor progression [202,203,204,205,206,207,208,209,210].

According to the classical multistep carcinogenesis model, accumulating multiple alterations of some of the above-mentioned molecular components are responsible for TC heterogeneity and the transition from well differentiated normal thyrocytes to well differentiated TC subtypes and finally, to most undifferentiated ATCs. Recently, an alternative model named “fetal/stem cell carcinogenesis hypothesis” has been proposed [211]. According to this new model, mutations or epigenetic alterations of normal thyroid adult stem cells or their committed progenitors present within the thyroid gland, induce their malignant transformation toward specific TC stem cells (TCSC), which, in turn, become the potential origin of distinct TC histotypes and the cells responsible for tumor progression, therapeutic resistance and recurrence [212]. Therefore, this last model regards the thyroid carcinogenesis process as an abnormal development of fetal-like thyroid cells, instead of de-differentiation of normal thyrocytes [213]. Preclinical data have shown that several pathways regulating self-renewal, proliferation and differentiation abilities are deregulated in TCSC. Alterations in the insulin/IGF system components, including increased expression of IR-A, IGF-1R, and IGF-2, and as a consequence, over-activation of the IR-A/IGF-2 autocrine loop have been found in TC stem/progenitor cells derived from PDTCs [214]. These results suggest that the IGF system may also be involved in follicular thyroid precursor regulation and biology. Other well-studied molecular alterations present in TCSCs include *RET/PTC* and *Pax8/PPAR*-γ rearrangements as well as deregulation in the MAPK pathway or Wnt/β-catenin, NOTCH, Hedgehog, JAK/STAT3 and NFkB pathways [214,215,216,217]. Furthermore, TCSCs obtained from undifferentiated thyroid carcinoma show constitutive activation of AKT, MET, and β-catenin and loss of E-cadherin, TWIST and SNAIL. MET or AKT targeting repressed the migration and metastatic behavior of thyroid stem cells as well as the expression of TWIST and SNAIL. These data suggest a role for AKT, MET, β-catenin and the IGF system in mediating an aggressive metastatic phenotype of cancer stem cells that is consistent with that shown by PDTCs [214,218].

Recent evidence suggests that also non-coding RNAs, both microRNAs (miRNAs) and long-non coding RNAs (lncRNAs), may play a role in thyroid carcinogenesis due to their ability to modulate target genes involved in several pathological pathways and biological processes such as differentiation, proliferation, apoptosis, and stemness [219,220,221]. Sheng et al. have identified miRNA-148a and its target *INO80* as crucial regulators of the proliferative and tumor-forming capacity of ATC-CSCs [222]. In another study, the antisense-mediated downregulation of miR-21 has been seen to enhance differentiation and apoptosis and to reduce cancer stemness features and cell cycle progression of ATC cells [223]. However, lncRNA-H19 was found highly expressed in cancer stem cells from PTCs where its depletion significantly reversed E2-induced sphere formation capability and stem-like properties [224]. Similarly, LIN00311 was found upregulated in PTC tissues and cells, where it promoted cancer stem-like properties by targeting miR-330-5p/TLR4 pathway [225].

Although many aspects of thyroid cancer initiation and progression still remain unclear, the discovery of TCSCs and signals regulating their biology may provide new insight into the pathologic mechanism of thyroid tumorigenesis and may open new perspectives in terms of prevention, diagnosis and therapy. Indeed, targeting TCSCs and/or the signaling pathways and/or the factors involved in their self-renewal, proliferation and differentiation abilities may contribute to overcome the resistance to anti-cancer therapies and achieve long-lasting remission.

## 4. Circadian Clock and Thyroid Tumorigenesis

A large body of evidence has suggested that different components and functions of the endocrine system, including the hypothalamic-pituitary-thyroid axis, the rhythmicity of TSH and of thyroid hormones secretion, are driven not only by behavior-associated factors, but also by an intrinsic timekeeping machinery, including the central hypothalamic clock as well as peripheral clocks [226,227]. The connection between circadian clock and thyroid function is reciprocal. The circadian and ultradian TSH rhythm, the daily rhythmicity of circulating thyroid hormones T4, Free T4 and T3 are influenced by sleep-wake homeostasis [21,23,228,229,230].

In turn, thyroid hormone deficiency or excess may affect the expression of core clock genes and metabolic clock-controlled genes in several peripheral tissues [231,232,233,234]. Similarly to most cells of the body, a rhythm-generating circuitry composed of a number of clock genes and several autoregulatory feedback loops has also been revealed in cultured human primary thyrocytes derived from healthy thyroid tissue [8]. In support of the existence of a thyroid clock, circadian oscillations for core clock genes have been demonstrated in rats as well as in in vitro synchronized human primary thyrocytes, which present a circadian period length of about 27 h [8,228]. Different studies, although sometimes with conflicting results, have demonstrated a possible relationship between circadian clockwork and thyroid tumorigenesis [5,6,7,8,9,10].

Insulin resistance could represent a plausible biological link for this association. Indeed, insulin resistance has been implicated in TC development and progression and it is often increased upon circadian clock disruption. Furthermore, this metabolic alteration is associated with an elevation of serum TSH level, which is, in turn, a well-known risk factor for TC [19,20] and is also increased upon sleep-wake cycle disturbances [21,235,236]. However, to our knowledge, clinical studies conducted to better understand this relationship are not available to date. Furthermore, the studies regarding the association between sleep disorders and risk of TC do not help, because they have often been contradictory. Indeed, two cohort studies conducted in flight attendants and flight crews did not support this association [237,238]. Conversely, a large prospective study has indicated that postmenopausal women affected by sleep disorders showed a significantly increased risk of TC (HR = 1.44), which was surprisingly limited to non-obese subjects (HR = 1.71) and was not seen in obese women (HR = 0.94) [239]. These contradictory results suggest that additional clinical studies with sufficient sample size and strong statistical power are urgently needed to apply and validate these findings on a larger population. Several in vitro studies have tried to answer some questions in order to confirm the connection between circadian clocks and TC transformation and to better characterize the possible biological mechanisms underlying this association.

SNPs or deregulation of several clock genes including *PER1-2-3, CRYs, REV-ERBα−β* and *RORα−β−γ* have recently been found associated with a higher risk of TC [14,240]. Increased expression levels of the circadian clock factor Differentially Expressed in Chondrocyte 1 (DEC1), has been implicated in TC promotion by the induction of several cell-cycle-related genes [241]. Up-regulation of BMAL1 and downregulation of CRY2 have been observed in tissue samples from FTC and PTC nodule tissues compared to benign tissues, which show functional circadian oscillators. Endogenous transcript analysis of primary thyrocytes established from PDTCs revealed a robust disruption of circadian gene expression [8]. In particular, PER1 transcripts showed ablated circadian amplitude, whereas BMAL1, PER2/3 and REV-ERBα displayed a strong phase shift compared to thyrocytes established from benign nodules. Similar results were obtained using a long-term continuous circadian bioluminescence oscillation monitoring by transducing BMAL1–luciferase lentivectors into healthy, benign nodules and PDTC-PTC-derived thyrocytes. These last types of cells showed a robust shifted or even anti-phasic pattern of BMAL1-luc reporter oscillatory expression compared to healthy and benign tissue counterparts [8]. These results suggest that the circadian clock machinery is altered upon thyroid malignant transformation. Similar findings were recently confirmed by a Nanostring approach in PTC, FTC, and PDTC tissue samples, which showed significant alterations in core clock genes (*BMAL1* and *CRY2*) and in other genes related to the cell-cycle and apoptosis [5,7]. In particular, *PER2* core clock transcript level was found downregulated in oncocytic FTCs and in PDTCs; *CRY2* was significantly downregulated in PTCs and PDTCs, while *BMAL1* was upregulated in PTCs compared to normal thyroid and benign nodules [5,7]. Based on these alterations in gene expression, a correlation coefficient for the diagnosis of FTCs has been proposed [7]. Furthermore, distinct molecular profiles of key components of clock machinery, cell cycle, apoptosis and Wnt signaling were observed for oncocytic and non-oncocytic FTCs and PDTCs. The more aggressive oncocytic subgroups showed higher numbers of altered genes compared to their non-oncocytic counterparts, revealing that alteration levels of several transcripts might correlate to tumor progression [7]. In line with these data, another study reported an altered expression of *REV-ERB*α and *ROR*α genes in PTCs especially in those positive for *BRAF*-mutation [242].

**Figure 2:**
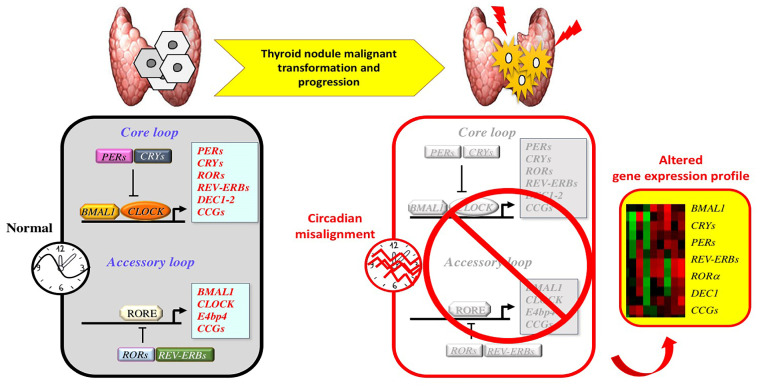
Molecular alterations in circadian clock gene machinery during thyroid nodule malignant transformation and progression. The circadian transcriptional/translation machinery physiologically acts through a core loop in which *CLOCK/BMAL1* activate transcription by binding E-boxes in the promoters of target genes (*PERs, CRYs, REV-ERBs*, *RORs, DECs, WEE1, c-MYC* and other clock-controlled genes (*CCGs*)). In the same loop, the negative PERs and CRYs proteins multimerize and inhibit *CLOCK/BMAL1* activity. Clock machinery is also regulated by an accessory loop, consisting of antagonizing transcription factors such as REV-ERBs (α−β) and RORs (α−β−γ), which regulate CLOCK/*BMAL1* gene expression and *CLOCK/BMAL1*-mediated *CCGs* transcription through ROR-elements (RORE). During thyroid tumorigenesis, circadian misalignment is associated with an altered expression of several clock genes and other *CCGs* controlling cellular and metabolic functions. These molecular alterations may contribute to thyroid nodule malignant transformation and progression.

Overall these results suggest that circadian clock characteristics are altered upon thyroid nodule malignant transformation/progression and that changes in clock gene expression profiles may be potentially employed in clinics as potential biomarkers for FTCs and disease progression (Figure 2). Despite the fact that this attempt might represent a great potential for the preoperative diagnosis of TC, further preclinical and epidemiological studies are needed for a rigorous confirmation.

The name and functions of the main genes and corresponding proteins involved in circadian clock machinery regulation and thyroid tumorigenesis are listed in Table 1.

## 5. Conclusions

The connection between circadian clock machinery dysfunction and TC has different clinical implications in terms of TC prevention, diagnosis and therapy.

Firstly, circadian misalignment could represent a putative risk factor suspected to play a potential role in the changing epidemiology of TC. The increased incidence of TC is largely dependent on modifiable risk factors, such as environmental carcinogens, diet habits, insulin resistance, therapies and lifestyle modifications [243], which may include circadian misalignment. Sleep disturbances and disruption in circadian synchronization are spreading worldwide as a consequence of occupational and personal pressure [11,239].

Chronic disruption of the clockwork has long-term consequences on health becoming a risk factor for insulin resistance, type 2 diabetes mellitus, obesity, atherosclerosis, cardiovascular diseases and cancers including endocrine-dependent tumors [11,67,68,147,244,245].

A reciprocal connection between circadian clock and thyroid disorders has been described in both in vitro and in vivo studies. Chronic sleep deprivation has been associated with disruption of rhythmic TSH secretion, which, in turn, is linked to an increased incidence of human TC [19,20]. Furthermore, disruption of circadian rhythm has been linked to alterations in gene-related apoptosis, DNA damage, cell cycle, and stemness, and thereby to carcinogenesis [11,55,67,68,149,246]. However, some oncogenes such as *RAS*, which is implicated in thyroid tumorigenesis, induce dysregulation of circadian clocks in human cancer cell lines [6,247]. In light of this evidence, it is biologically plausible that circadian clock alterations could represent a potential risk factor of developing TC. However, so far, no epidemiologic study has been directly addressed in this relationship.

Another concept to be highlighted is that clock gene expression profile could be helpful to improve the pre-operative diagnostics of thyroid nodules, especially those cytologically indeterminate or with a follicular pattern. Alterations in the expression profiles of clock genes (i.e. *BMAL1, CRYs, REV-ERBα* and *PERs*) and of cell cycle key components have recently been observed in both PTCs and FTCs when compared to benign nodules or healthy tissue [5,7,8]. Based on these distinct molecular expression profiles a predictive score correlation coefficient with high sensitivity and specificity has been proposed to distinguish between FTCs and benign follicular lesions [7]. The potential use of clock gene expression profiling as predictive markers of TC provides new insights into the molecular mechanisms underlying the pathophysiology of malignant thyroid nodules giving important perspectives in scientific and clinical fields. However, there is an urgent need to launch large prospective studies to confirm this preclinical evidence.

Last but not least, the synchronization of circadian rhythm and/or targeting clock gene alterations starting from TC progenitor cells may represent new adjunct therapeutic strategies to improve the clinical management of TCs especially those developed in insulin resistant patients with circadian clock disruption. Hyperinsulinemia, present in insulin resistant conditions, may worsen the prognosis of TC likely by potentiating IR-A/IGF2-dependent mitogenic functions. Dysfunction of circadian timing leads to an increased risk of insulin resistance-related metabolic disorders [11,245,248,249,250]. Conversely, pharmacological treatments enhancing circadian rhythm or chrono-pharmacology exert beneficial effects on metabolic fitness [16,17]. Based on these observations, it is reasonable to expect that improving insulin resistance through synchronization of circadian rhythm or chronotherapy in conjunction with a healthy diet, physical activity and conventional anti-cancer therapies, could exert beneficial effects on prevention and treatment of TCs developed in insulin resistant patients with disrupted circadian rhythms. However, to date, studies aimed at evaluating the efficacy of all these therapeutic options as an add-on therapy for patients with TCs in the context of insulin resistance and circadian misalignment are lacking.

## Figures and Tables

**Table 1:** List describing in alphabetic order name, major putative functions/signaling pathways of genes and corresponding products involved in circadian clock machinery regulation and thyroid tumorigenesis.

Gene Name	Protein Name	Function/Signaling Pathway
*AKT* or *PKB (Protein kinase B)*	Protein kinase B (PKB)	Survival, proliferation, apoptosis resistance. PI3K/AKT signaling pathway
*ATM (ataxia telangiectasia)*	ATM	DNA damage response, cell cycle, apoptosis, mitochondrial homeostasis
*ATR (ataxia telangiectasia and Rad-3 related protein)*	ATR	DNA damage response, cell cycle. PI3K/AKT signaling pathway
*BMAL1 (aryl hydrocarbon receptor nuclear translocator like)*	BMAL1	Circadian clock, exercise-induced circadian regulation, melatonin metabolism and effects, bone metabolism, energetic metabolism, cell stress
*BRAF (B-Raf proto-oncogene, serine/threonine kinase)*	BRAF	Oncogene. Proliferation, differentiation. MAPK/ERK signaling pathway
*c-MYC*	C-MYC	Proto-oncogene, transcription factor. Cell growth, apoptosis, differentiation, stem cell self-renewal.
*CK1 (casein kinase 1)*	CK1α-β-γ-δ-ε casein kinase 1α-β-γ-δ-ε)	Tumor suppressor. Circadian clock, metabolism, DNA damage, cellular stress, cell cycle, cytoskeleton associated functions. Developmental pathways
*CLOCK (clock circadian regulator)*	CLOCK	Circadian clock, exercise-induced circadian regulation, melatonin metabolism and effects.
*CRYs (cryptochrome circadian regulators)*	CRY1-2	Tumor suppressor. Circadian clock.
*DEC1-2 (differentially expressed in chondrocytes 1-2)*	DEC1-2	Tumor suppressor. Circadian clock.
*INO80*	Chormatin-remodeling ATPase INO80	Cell cycle, cell division, DNA damage, DNA recombination, DNA repair, mitosis, chromatin remodeling.
*MEN1*	Menin	Transcriptional regulator. Telomerase repressor. Cell proliferation, DNA repair. TGFB1 and NFkB signaling
*MET*	Proto-oncogene c-Met	Proliferation, scattering, morphogenesis, survival, differentiation, angiogenesis. RAS/ERK, PI3K/AKT, PLC-γ/PKC signaling
*NF1 (neurofibromatosis Type 1 Protein)*	Neurofibromin 1	Tumor suppressor. Cell growth and division. Ras inhibition. Circadian clock.
*NPAS2 (neuronal PAS domain protein 2)*	NPAS2	Tumor suppressor. DNA damage response. Negative regulator of cell death. Circadian clock. Central nervous system development. Metabolism.
*P53*	P53	Tumor suppressor. Response to DNA damage. Cell cycle arrest. Apoptosis. Aging. Gene expression
*PI3KCA (phosphatidylinositol-4,5-bisphosphate 3-kinase 110 kDa catalytic subunit alpha)*	Phosphatidylinositol-4,5-bisphosphate 3-kinase catalytic subunit alpha isoform	Catalytic activity (performs the action of PI3K). Proliferation, survival, migration. PI3K/AKT/mTOR signaling pathway.
*PER1-2-3 (Period circadian regulator 1-2-3)*	PER1-2-3	Tumor suppressor. Circadian clock. Exercise-induced circadian regulation. Melatonin metabolism and effects. Chromatin DNA binding
*PTEN (phosphatase and tensin homolog)*	PTEN	Tumor suppressor. AKT/PKB signaling pathway
*RAS*	RAS	Oncogene. Cell growth, differentiation, survival, Cell adhesion, apoptosis, migration. MAPK/ERK and PI3K/AKT/mTOR pathway
*RB1 (RB transcriptional corepressor 1)*	Retinoblastoma associated protein RB1	Tumor suppressor. Cell cycle. Chromatin remodeling. Cell differentiation, cell growth
*REV-ERB or NR1D1 (nuclear receptor subfamily 1 group D member 1)*	NR1D1	Tumor suppressor. Circadian clock. Mitochondrial biogenesis. Nuclear Receptor transcription pathway
*RET (RET proto-oncogene)*	RET	Proto-oncogene. Protein tyrosine kinase activity. MAPK signaling.
*RORα (RAR related orphan receptor A)*	Nuclear receptor ROR-alpha	Tumor suppressor. Circadian clock. Metabolism. Transcription factor activity.
*SWI/SNF (SMARCC1)*	SWI/SNF complex subunit SMARCC1	Chromatin remodeling. Transcription regulator
*TERT (telomerase reverse transcriptase)*	Telomerase reverse transcriptase	Chromosome replication. Telomerase activity. Transcription regulator
*TIM (timeless circadian regulator)*	Protein timeless homolog, hTIM	Tumor suppressor. Circadian clock. DNA replication, replication fork stability
*WEE1*	WEE1-like protein kinase	Cell division, cell cycle, microtubule cytoskeleton organization

## Abbreviations

ATC	Anaplastic Thyroid Cancer
ATM	Ataxia Telangiectasia Mutated
ATR	Ataxia Telangiectasia and Rad3-related protein
CCGs	Clock-Controlled Genes
CCSC	Colon Cancer Stem-like Cells
DBP	D-box-binding protein
DDR1	Discoidin Domain Receptor 1
DEC1	Differentially Expressed in Chondrocyte 1
DKO	Double knockout
ES	Embryonic stem cells
FNA	Fine-Needle Aspiration
FTC	Follicular Thyroid Cancer
GSC	Glioma Stem Cells
HAT	Histone acetyltransferase
HCC	Hurthle Cell Carcinoma
HGF	Hepatocyte Growth Factor
HPT	Hypothalamic-Pituitary-Thyroid
IGF-1R	Insulin-like growth factor-1 receptor
IGF-2	Insulin-like growth factor-2
IGF	Insulin-like growth factor
KO	Knockout
lncRNAs	Long-non coding RNAs
miRNAs	MicroRNAs
PAX8/PPAR	Paired-box gene 8/Peroxisome Proliferator-Activated Receptor gamma
PDTC	Poorly Differentiated Thyroid Cancer
PKB	Protein Kinase B
PTC	Papillary Thyroid Cancer
SCN	Suprachiasmatic Nucleus
TC	Thyroid Cancer
TCSC	Thyroid Cancer Stem Cell
TSH	Thyroid-Stimulating Hormone
